# Review of “Contemporary computer-assisted approaches to molecular structure elucidation (new developments in NMR)” by Mikhail E Elyashberg, Antony Williams and Kirill Blinov

**DOI:** 10.1186/1758-2946-5-29

**Published:** 2013-06-03

**Authors:** Christoph Steinbeck

**Affiliations:** 1 European Molecular Biology Laboratory - European Bioinformatics Institute (EMBL-EBI), Hinxton, UK

## Book details

Contemporary Computer-Assisted Approaches to Molecular Structure Elucidation (New Developments in NMR) by Mikhail E Elyashberg, Antony Williams and Kirill Blinov. Edited by William Price. RSC Publishing, 2012. ISBN: 978-1-84973-432-5; eISBN: 978-1-84973-457-8.

Computer-assisted structure elucidation (CASE) aims to provide users in chemistry, molecular biology, or other areas dealing with structures of small molecules with suggestions on the structural identity of molecules based on spectroscopic, chromatographic and other boundary information. With "Contemporary computer-assisted approaches to molecular structure elucidation", Mikhail Elyashberg, Antony Williams and Kirill Blinov, all world-renowned experts on the topic, have recently written a normative standard text-book on the topic. Published by RSC publishing in 2012 and on 481 pages, the book provides a comprehensive overview on computer-assisted structure elucidation.

The book is divided into three parts comprised of overall 14 chapters.

Part I lays out the fundamentals of CASE systems: the authors walk the reader through chapters covering different possible strategies for computer-assisted structure elucidation after which they give a brief history of the field. As the authors point out, the CASE process can be reduced to logically interfering ‘the most probable structural hypothesis from a set of statements reflecting the interrelation between a spectrum and structure’. Given that every human expert in structure elucidation will be biased by his or her own education, history of certain compound classes worked on, or types of spectroscopy used, CASE systems can be incredibly valuable by suggesting solutions to the structure elucidation problem outside of our range of experience. In chapter 2, the authors describe a set of fundamental assumptions about the interpretation of spectroscopic data that are typically made in both the human as well as the machine-based structure or sedation process, followed by an account of methods of NMR spectrum prediction and structure verification in chapter 3. The following chapter 4 on methods of relative stereochemistry determination in CASE systems concludes part number one. The second part of the book is dedicated to a walk-through of examples of existing case expert systems that have bullied been produced and published in the last 40 years. The last part of the book, covering more than half of the total number of pages, is then dedicated to the structure elucidation expert system developed by the authors themselves called "Structure Elucidator". This is certainly justified, because structure elucidator is easily the most comprehensive and usable case system on the market to date. To the best of my knowledge, there is no system even close to structure elucidator in terms of coverage, comprehensiveness, usability and speed. In this final chapter the authors describe the fundamental knowledge bases underlying the structure elucidator system, followed by an account of the primary data processing strategies within structure elucidator, after which they turn to the approaches used for structure elucidation in the system. In this chapter they provide interesting examples of a number of complex natural products that can be elucidated it with this system. Natural product structure elucidation and the revision of already published natural product structures are the topic of the following chapters. The book concludes with two chapters on the comparison of a systematic case system approach to the traditional approach to structure elucidation as well as an evaluation of the performance of the structure elucidator system.

The book delivers what the title promises. I have been working on CASE systems in the last 15 years and wrote a number of reviews on the topic. This is clearly the most comprehensive book on computer-assisted structure elucidation on the market and generally a valuable resource for anyone working in the area of structure elucidation of small molecules.

## Competing interests

The author declares that he has no competing interests.

